# Supplementary material for: Mediators of Lifestyle Behaviour Changes in Obese Pregnant Women. Secondary Analyses from the DALI Lifestyle Randomised Controlled Trial
Source: Nutrients. 2019 Feb 1;11(2):311. doi: 10.3390/nu11020311 (PMC6412227; doi:10.3390/nu11020311)
Supplement: Supplementary file 1 [file nutrients-11-00311-s001.docx]

**Table S1. List of statements for assessing outcome expectancy, task self-efficacy and perceived social support.**

| 1. **Attitude weight:** | - **I am bothered with my current weight** - **It is important for me to manage my weight** |
| --- | --- |
| 1. Outcome expectancy weight management for reducing GDM risk: | - Managing my weight during this pregnancy, will help me to reduce *my risk of developing GDM* |
| 1. Outcome expectancy physical activity for reducing GDM risk: | - Staying physically active during this pregnancy, will help me to reduce *my risk of developing GDM* |
| 1. Outcome expectancy healthy eating for reducing GDM risk: | - Eating healthily during this pregnancy, will help me to reduce *my risk of developing GDM* |
| 1. Outcome expectancy weight management for reducing risk for the baby: | - Managing my weight during this pregnancy, will help to reduce *health risks for my baby* |
| 1. Outcome expectancy physical activity for reducing risk for the baby: | - Staying physically active during this pregnancy, will help to reduce *health risks for my baby* |
| 1. Outcome expectancy healthy eating for reducing risk for the baby: | - Eating healthily during this pregnancy, will help to reduce *health risks for my baby* |
| 1. Task self-efficacy weight: | - I am confident that I will succeed in managing my weight |
| 1. Task self-efficacy healthy eating: | - I am confident that I will succeed in eating healthily on a daily basis right now - I am confident that I will succeed in eating healthily on a daily basis in the coming weeks |
| 1. Task self-efficacy physical activity: | - I am confident that I can be physically active on a daily basis right now - I am confident that I can be physically active on a daily basis as planned in the coming weeks |
| 1. Satisfaction with social support for physical activity: | - I am satisfied with the level of support I am receiving for being physically active from my partner, family and friends |
| 1. Satisfaction with social support for healthy eating: | - I am satisfied with the level of support I am receiving for eating healthily from my partner, family and friends |

B&C and E&F were combined for outcome expectancy in relation to moderate-to-vigorous physical activity (MVPA) and sedentary behaviour, B&D and E&G were combined for outcome expectancy in relation to dietary outcomes. H&J were combined for task self-efficacy in relation to MVPA and sedentary behaviour, H&I were combined for task self-efficacy in relation to dietary outcomes.

**Table S2a. Moderate to vigorous physical activity** **at 24-28 weeks and 35-37 weeks, mediators at 24-28 weeks.**

| **MVPA at 24-28 weeks (*n* = 382)** | **Effect of mediator on MVPA (b-path)** | Indirect effect (path a*b) | | |
| --- | --- | --- | --- | --- |
|  | **Estimate (95% CI)** | HE&PA  Estimate (95% CI) | HE  Estimate (95% CI) | PA  Estimate (95% CI) |
| Outcome expectancy GDM risk reduction | -0.001 (-0.05; 0.05) | -0.002 (-0.09; 0.08) | -0.001 (-0.09; 0.08) | -0.002 (-0.09; 0.09) |
| Outcome expectancy risk reduction baby | -0.007 (-0.06; 0.05) | -0.01 (-0.09; 0.05) | -0.01 (-0.09; 0.06) | -0.01 (-0.09; 0.06) |
| Task self-efficacy | **0.04 (0.02; 0.07)** | **0.09 (0.03; 0.19)** | 0.04 (-0.07; 0.12) | **0.08 (0.02; 0.18)** |
| Satisfaction with social support | -0.01 (-0.07; 0.04) | -0.01 (-0.05; 0.03) | -0.005 (-0.05; 0.02) | -0.01 (-0.05; 0.02) |
| Perceived risk for GDM | -0.05 (-0.17; 0.06) | -0.001 (-0.03; 0.01) | 0.003 (-0.02; 0.02) | -0.002 (-0.02; 0.02) |
| MVPA at 35-37 weeks (*n* = 332) |  | HE&PA | HE | PA |
| Outcome expectancy GDM risk reduction | -0.01 (-0.08; 0.07) | -0.01 (-0.15; 0.10) | -0.01 (-0.13; 0.10) | -0.01 (-0.16; 0.12) |
| Outcome expectancy risk reduction baby | -0.02 (-0.09; 0.06) | -0.01 (-0.15; 0.05) | -0.02 (-0.15; 0.07) | -0.02 (-0.15; 0.08) |
| Task self-efficacy | **0.03 (0.02; 0.06)** | **0.06 (0.01; 0.16)** | 0.03 (-0.01 0.11) | **0.08 (0.01; 0.20)** |
| Satisfaction with social support | 0.02 (-0.06; 0.10) | 0.01 (-0.02; 0.07) | 0.01 (-0.01; 0.06) | 0.01 (-0.01; 0.07) |
| Perceived risk for GDM | 0.001 (-0.15; 0.15) | 0.0001 (-0.03; 0.03) | 0.000 (-0.02; 0.02) | 0.000 (-0.02; 0.02) |

Abbreviations: CI= Confidence Interval; HE=Healthy Eating; PA=Physical Activity; HE&PA= Healthy Eating & Physical Activity; GDM=gestational diabetes; MVPA=moderate-to-vigorous physical activity.

**Table 2Sb. Sedentary behaviour at at 24-28 weeks and 35-37 weeks, mediators at 24-28 weeks 3.**

| Sedentary behaviour at 24-28 weeks (*n* = 387) | Effect of mediator on sedentary behaviour (b-path) | Indirect effect (path a*b) | | |
| --- | --- | --- | --- | --- |
|  | Estimate (95% CI) | HE&PA  Estimate (95% CI) | HE  Estimate (95% CI) | PA  Estimate (95% CI) |
| Outcome expectancy GDM risk reduction | -0.16 (-0.59; 0.26) | -0.21 (-1.04; 0.27) | -0.20 (-0.93; 0.27) | -0.22 (-1.01; 0.30) |
| Outcome expectancy risk reduction baby | 0.21 (-0.24; 0.65) | 0.17 (-0.12; 0.96) | 0.19 (-0.14; 0.89) | 0.20 (-0.15; 0.86) |
| Task self-efficacy | -0.18 (-0.37; 0.01) | -0.37 (-1.11; 0.03) | -0.19 (-0.78; 0.03) | -0.33 (-1.05; 0.02) |
| Satisfaction with social support | -0.18 (-0.65; 0.29) | -0.10 (-0.60; 0.19) | -0.07 (-0.52; 0.12) | -0.09 (-0.54; 0.15) |
| Perceived risk for GDM | 0.36 (-0.56; 1.28) | 0.01 (-0.07; 0.26) | 0.001 (-0.13; 0.18) | -0.004 (-0.19; 0.10) |
| Sedentary behaviour at 35-37 weeks (*n* = 336) |  | HE&PA | HE | PA |
| Outcome expectancy GDM risk reduction | 0.02 (-0.53; 0.57) | 0.03 (-0.78; 0.82) | 0.02 (-0.76; 0.70) | 0.03 (-0.89; 0.83) |
| Outcome expectancy risk reduction baby | 0.001 (-0.55; 0.55) | 0.001 (-0.54; 0.62) | 0.002 (-0.65; 0.68) | 0.002 (-0.67; 0.68) |
| Task self-efficacy | -0.14 (-0.36; 0.09) | -0.25 (-0.80; 0.11) | -0.13 (-0.62 0.07) | -0.31 (-1.03; 0.14) |
| Satisfaction with social support | -0.02 (-0.60; 0.57) | -0.01 (-0.44; 0.35) | -0.01 (-0.31; 0.23) | -0.01 (-0.34; 0.25) |
| Perceived risk for GDM | 0.14 (-0.95; 1.23) | 0.01 (-0.09; 0.30) | 0.003 (-0.12; 0.19) | -0.003 (-0.19; 0.12) |

Abbreviations: CI= Confidence Interval; HE=Healthy Eating; PA=Physical Activity; HE&PA= Healthy Eating & Physical Activity; GDM=gestational diabetes;

**Table S2c. Sugared drink consumption at at 24-28 weeks and 35-37 weeks, mediators at 24-28 weeks**

|  | **Effect of mediator on sugared drink consumption (b-path)** | Indirect effect (path a*b) | | |
| --- | --- | --- | --- | --- |
| **Sugared drink consumption at 24-28 weeks (*n* = 354)** | **Estimate (95% CI)** | HE&PA  Estimate (95% CI) | HE  Estimate (95% CI) | PA  Estimate (95% CI) |
| Outcome expectancy GDM risk reduction | -0.25 (-0.66; 0.17) | -0.31 (-1.10; 0.25) | -0.31 (-1.05; 0.25) | -0.27 (-0.99; 0.18) |
| Outcome expectancy risk reduction baby | 0.40 (-0.04; 0.85) | **0.38 (0.02; 1.13)** | **0.38 (0.01; 1.21)** | **0.43 (0.02; 1.32)** |
| Task self-efficacy | **-0.24 (-0.42; -0.06)** | **-0.56 (-1.43; -0.07)** | **-0.48 (-1.27; -0.06)** | -0.13 (-0.69; 0.11) |
| Satisfaction with social support | -0.11 (-0.57; 0.36) | -0.02 (-0.31; 0.07) | -0.04 (-0.34; 0.11) | -0.01 (-0.23; 0.07) |
| Perceived risk for GDM | -0.32 (-1.11; 0.46) | -0.01 (-0.23; 0.07) | -0.005 (-0.17; 0.10) | -0.004 (-0.16; 0.09) |
| Sugared drink consumption at 35-37 weeks (*n* = 295) |  | HE&PA | HE | PA |
| Outcome expectancy GDM risk reduction | 0.05 (-0.63; 0.74) | 0.08 (-0.86; 1.31) | 0.07 (-0.69; 1.19) | 0.08 (-0.78; 1.28) |
| Outcome expectancy risk reduction baby | 0.05 (-0.66; 0.76) | 0.06 (-0.79; 1.09) | 0.06 (-0.76; 1.07) | 0.07 (-0.86; 1.21) |
| Task self-efficacy | -0.24 (-0.51; 0.04) | -0.63 (-1.90; 0.20) | -0.54(-1.75; 0.15) | -0.29(-1.29; 0.07**)** |
| Satisfaction with social support | -0.17 (-0.93; 0.58) | -0.06 (-0.58; 0.14) | -0.08(-0.64; 0.24) | -0.02 (-0.43; 0.10) |
| Perceived risk for GDM | -0.58 (-1.79; 0.63) | -0.05 (-0.59; 0.07) | -0.03 (-0.50; 0.09) | -0.004 (-0.31; 0.19) |

Abbreviations: CI= Confidence Interval; HE=Healthy Eating; PA=Physical Activity; HE&PA= Healthy Eating & Physical Activity; GDM=gestational diabetes; MVPA=moderate-to-vigorous physical activity.

**Table S2d. Vegetable consumption at 24-28 weeks and 35-37 weeks, mediators at 24-28 weeks**

|  | **Effect of mediator on vegetable consumption (b-path)** | Indirect effect (path a*b) | | |
| --- | --- | --- | --- | --- |
| **vegetable consumption at 24-28 wks (*n* = 357)** | **Estimate (95% CI)** | HE&PA  Estimate (95% CI) | HE  Estimate (95% CI) | PA  Estimate (95% CI) |
| Outcome expectancy GDM risk reduction | 0.42 (-0.11; 0.95) | 0.53 (-0.04; 1.42) | 0.52 (-0.04; 1.44) | 0.47 (-0.02; 1.38) |
| Outcome expectancy risk reduction baby | -0.22 (-0.79; 0.35) | -0.20 (-0.96; 0.23) | -0.20 (-0.04; 1.44) | -0.21 (-0.94; 0.27) |
| Task self-efficacy | 0.15 (-0.08; 0.39) | 0.34 (-0.15; 1.04) | 0.36 (-0.17; 1.07) | 0.08 (-0.08; 0.61) |
| Satisfaction with social support | -0.28 (-0.88; 0.32) | -0.03 (-0.40; 0.10) | -0.11 (-0.60; 0.08) | -0.02 (-0.35; 0.11) |
| Perceived risk for GDM | 0.73(-0.30; 1.77) | 0.06 (-0.08; 0.46) | 0.02 (-0.14; 0.37) | -0.01 (-0.27; 0.18) |
| vegetable consumption at 35-37 weeks (*n* = 303) |  | HE&PA | HE | PA |
| Outcome expectancy GDM risk reduction | -0.21 (-0.95; 0.52) | -0.36 (-1.85; 0.66) | -0.30 (-1.68; 0.56) | -0.31 (-1.60; 0.56) |
| Outcome expectancy risk reduction baby | 0.47 (-0.32; 1.25) | 0.57 (-0.18; 1.98) | 0.52 (-0.18; 1.91) | 0.56 (-0.17; 1.94) |
| Task self-efficacy | 0.01 (-0.30; 0.32) | 0.03 (-0.85; 0.81) | 0.03 (-0.84 0.79) | 0.01 (-0.32; 0.47**)** |
| Satisfaction with social support | -0.03 (-0.88; 0.83) | -0.004 (-0.36; 0.26) | -0.01 (-0.50; 0.45) | 0.001 (-0.22; 0.27) |
| Perceived risk for GDM | 0.03 (-1.33; 1.39) | 0.004 (-0.24; 0.29) | 0.002 (-0.18; 0.36) | 0.000 (-0.18; 0.19) |

Abbreviations: CI= Confidence Interval; HE=Healthy Eating; PA=Physical Activity; HE&PA= Healthy Eating & Physical Activity; GDM=gestational diabetes; MVPA=moderate-to-vigorous physical activity.

**Table S2e. Carbohydrate consumption at 24-28 weeks and 35-37 weeks, mediators at 24-28 weeks**

|  | **Effect of mediator on carbohydrate consumption (b-path)** | Indirect effect (path a*b) | | |
| --- | --- | --- | --- | --- |
| **carbohydrate consumption at 24-28 weeks (*n* = 328)** | **Estimate (95% CI)** | HE&PA  Estimate (95% CI) | HE  Estimate (95% CI) | PA  Estimate (95% CI) |
| Outcome expectancy GDM risk reduction | -0.03 (-1.09; 1.02) | -0.05 (-1.97; 1.76) | -0.05 (-1.90; 1.63) | -0.03 (-1.30; 1.12) |
| Outcome expectancy risk reduction baby | 0.16 (-0.99; 1.32) | 0.16 (-1.00; 1.63) | 0.17 (-1.04; 1.72) | 0.12 (-0.69; 1.42) |
| Task self-efficacy | -0.19 (-0.66; 0.27) | -0.43 (-1.83; 0.69) | -0.45 (-1.87; 0.70) | -0.05 (-0.84; 0.24) |
| Satisfaction with social support | -0.20 (-1.41; 1.01) | -0.05 (-0.77; 0.22) | -0.08 (-0.82; 0.38) | 0.004 (-0.30; 0.36) |
| Perceived risk for GDM | -1.41 (-3.39; 0.56) | -0.11 (-0.88; 0.17) | -0.03 (-0.61; 0.32) | -0.05 (-0.62; 0.27) |
| carbohydrate consumption at 35-37 weeks (*n* = 270) |  | HE&PA | HE | PA |
| Outcome expectancy GDM risk reduction | 0.04 (-1.91; 1.99) | 0.07 (-2.96; 2.98) | 0.06 (-2.41; 2.53) | 0.05 (-2.20; 2.22) |
| Outcome expectancy risk reduction baby | 0.47 (-1.59; 2.54) | 0.62 (-1.20; 3.57) | 0.57 (-1.15; 3.50) | 0.48 (-0.91; 2.97) |
| Task self-efficacy | -0.17 (-0.95; 0.61) | -0.43 (-2.56; 1.44) | -0.43 (-2.56 1.50) | -0.13 (-1.39; 0.39**)** |
| Satisfaction with social support | -0.65 (-2.89; 1.60) | -0.26 (-2.05; 0.49) | -0.37 (-2.31; 0.79) | -0.02 (-0.98; 0.51) |
| Perceived risk for GDM | -2.02 (-5.47; 1.44) | -0.19 (-1.70; 0.23) | -0.20 (-1.66; 0.21) | -0.01 (-0.88; 0.65) |

Abbreviations: CI= Confidence Interval; HE=Healthy Eating; PA=Physical Activity; HE&PA= Healthy Eating & Physical Activity; GDM=gestational diabetes; MVPA=moderate-to-vigorous physical activity.

**Table S2f. Portion size at 24-28 weeks and 35-37 weeks, mediators at 24-28 weeks**

|  | **Effect of mediator on portion size (b-path)** | Indirect effect (path a × b) | | |
| --- | --- | --- | --- | --- |
| **portion size at 24-28 weeks (*n* = 346)** | **Estimate (95% CI)** | HE&PA  Estimate (95% CI) | HE  Estimate (95% CI) | PA  Estimate (95% CI) |
| Outcome expectancy GDM risk reduction | -0.66 (-1.54; 0.21) | -0.83 (-2.62; 0.17) | -0.87 (-2.70; 0.17) | -0.64 (-2.23; 0.10) |
| Outcome expectancy risk reduction baby | 0.90 (-0.05; 1.86) | **0.80 (0.05; 2.47)** | **0.97 (0.07; 2.73)** | **0.84 (0.05; 2.53)** |
| Task self-efficacy | -0.26 (-0.66; 0.14) | -0.62 (-1.97; 0.40) | -0.63 (-1.96; 0.42) | -0.16 (-1.06; 0.14) |
| Satisfaction with social support | -0.67 (-1.66; 0.33) | -0.14 (-1.14; 0.13) | -0.27 (-1.35; 0.11) | -0.03 (-0.71; 0.26) |
| Perceived risk for GDM | -0.01 (-1.72; 1.70) | -0.004 (-0.22; 0.21) | -0.004 (-0.22; 0.19) | -0.0002 (-0.21; 0.19) |
| portion size at 35-37 weeks (*n* =293) |  | HE&PA | HE | PA |
| Outcome expectancy GDM risk reduction | -0.20 (-1.20; 0.81) | -0.31 (-2.11; 1.22) | -0.29 (-1.97; 1.18) | -0.24 (-1.75; 0.94) |
| Outcome expectancy risk reduction baby | 0.37 (-0.70; 1.45) | 0.41 (-0.66; 2.32) | 0.47 (-0.80; 2.46) | 0.42 (-0.68; 2.23) |
| Task self-efficacy | -0.39 (-0.81; 0.04) | -1.01 (-2.72; 0.09) | -1.00 (-2.71 0.10) | -0.41 (-1.62; 0.09) |
| Satisfaction with social support | **-1.15 (-2.30; -0.01)** | -0.35 (-1.46; 0.11) | **-0.61 (-1.93; -0.03)** | -0.10 (-0.98; 0.40) |
| Perceived risk for GDM | -0.15 (-2.02; 1.72) | -0.01 (-0.48; 0.20) | -0.02 (-0.47; 0.21) | 0.002 (-0.21; 0.29) |

Abbreviations: CI= Confidence Interval; HE=Healthy Eating; PA=Physical Activity; HE&PA= Healthy Eating & Physical Activity; GDM=gestational diabetes; MVPA=moderate-to-vigorous physical activity.

**Table 3S. Total (c-path) and direct (c’-path) intervention effects on lifestyle behaviours*.**

| **Total effect** | **HE&PA vs UC** | **HE vs UC** | **PA vs UC** |
| --- | --- | --- | --- |
| **24-28 weeks** | **Beta (95% CI)** | **Beta (95% CI)** | **Beta (95% CI)** |
| MVPA, MET-h/week** | **0.27 (0.02, 0.52)** | 0.05 (-0.20, 0.29) | **0.35 (0.10, 0.60)** |
| Sedentary behaviour, MET-h/week | **-2.46 (-4.51, -0.40)** | **-2.37 (-4.37, -0.36)** | **-2.09 (-4.13, -0.05)** |
| Sugary drinks, portions /week | -1.31 (-4.22, 1.59) | -1.67 (-3.41, 0.07) | 1.43 (-0.36, 3.22) |
| Vegetables, portions /week | **2.49 (0.20, 4.79)** | **3.71 (1.47, 5.94)** | 0.12 (-2.16, 2.39) |
| Carbohydrates, portions /week | -1.78 (-6.24, 2.68) | **-4.62 (-8.88, -0.35)** | -2.44 (-6.79, 1.91) |
| Portion size, portions /week | -1.25 (-5.12, 2.62) | **-5.48 (-9.19, -1.76)** | -1.49 (-5.29, 2.31) |
| **Total effect**  **35-37 weeks** |  |  |  |
| MVPA, MET-h/week** | 0.06 (-0.27, 0.38) | 0.04 (-0.28, 0.36) | 0.26 (-0.06, 0.58) |
| Sedentary behaviour, MET-h/week | **-3.49 (-5.88, -1.10)** | -1.12 (-3.48, 1.25) | -1.19 (-3.55, 1.17) |
| Sugary drinks, portions /week | **-3.19 (-5.89, -0.50)** | -1.45 (-4.09, 1.20) | 1.51 (-1.15, 4.18) |
| Vegetables, portions /week | 0.72 (-2.23, 3.68) | **3.28 (0.33, 6.23)** | -1.31 (-4.22, 1.59) |
| Carbohydrates, portions /week | -4.70 (-12.21, 2.80) | **-4.94 (-12.29,2.41)** | -1.15 (-8.43, 6.13) |
| Portion size, portions /week | -4.01 (-8.14, 0.12) | **-4.03 (-8.04, -0.02)** | -0.04 (-4.06,3.98) |
| **Direct effect**  **24-28 weeks** |  |  |  |
| MVPA, MET-h/week** | 0.20 (-0.06; 0.45) | 0.02 (-0.23; 0.26) | **0.29 (0.04; 0.54)** |
| Sedentary behaviour, MET-h/wk | -1.95 (-4.05; 0.14) | **-2.09 (-4.12; -0.07)** | -1.64 (-3.71; 0.44) |
| Sugary drinks, portions /week | -1.57 (-4.56; 1.42) | -1.23 (-2.98; 0.52) | 1.42 (-0.37; 3.21) |
| Vegetables, portions /week | 1.80 (-0.55; 4.14) | **3.11 (0.83; 5.39)** | -0.20 (-2.48; 2.09) |
| Carbohydrates, portions /week | -1.30 (-5.93; 3.33) | -4.18 (-8.59; 0.23) | -2.44 (-6.85; 1.98) |
| Portion size, portions /week | -0.46 (-4.43; 3.50) | **-4.68 (-8.48; -0.87)** | -1.50 (-5.32; 2.32) |
| **Direct effect**  **35-37 weeks** |  |  |  |
| MVPA, MET-h/week ** | 0.01 (-0.32; 0.34) | 0.03 (-0.29; 0.36) | 0.20 (-0.13; 0.53) |
| Sedentary behaviour, MET-h/week | **-3.27 (-5.73; -0.81)** | -1.01 (-3.42; 1.40) | -0.90 (-3.34; 1.54) |
| Sugary drinks, portions /week | -2.60 (-5.38; 0.19) | -0.91 (-3.61; 1.79) | 1.69 (-1.03; 4.40) |
| Vegetables, portions /week | 0.49 (-2.61; 3.60) | 3.05 (-0.01; 6.10) | -1.57 (-4.56; 1.42) |
| Carbohydrates, portions /week | -4.52 (-12.41; 3.38) | -4.57 (-12.22; 3.08) | -1.52 (-9.00; 5.96) |
| Portion size, portions /week | -2.73 (-6.96; 1.48) | -2.59 (-6.66; 1.49) | 0.29 (-3.73; 4.31) |

Abbreviations: CI= Confidence Interval; UC= Usual Care; HE=Healthy Eating; PA=Physical Activity; HE&PA= Healthy Eating & Physical Activity; GDM=gestational diabetes; MVPA=moderate-to-vigorous physical activity.* The total intervention effects result from the parallel mediation analyses, therefore may differ slightly from results presented previously [9]. ** LN of MVPA
